# Effect of Lead(IV) Acetate on Procoagulant Activity in Human Red Blood Cells

**DOI:** 10.5487/TR.2009.25.4.175

**Published:** 2009-12-30

**Authors:** Keun-Young Kim, Kyung-Min Lim, Jung-Hun Shin, Ji-Yoon Noh, Jae-Bum Ahn, Da-Hye Lee, Jin-Ho Chung

**Affiliations:** 12 Institute of Pharmaceutical Sciences, College of Pharmacy, Seoul National University, Seoul, 151-742 Korea; 22 Korea Advanced Institute of Science and Technology, Daejeon, Korea

**Keywords:** Lead, Pb^4+^, Red blood cell, Phosphatidylserine exposure, Microvesicle generation, Hemolysis

## Abstract

Lead (Pb) is a ubiquitously occurring environmental heavy metal which is widely used in industry and human life. Possibly due to a global industrial expansion, recent studies have revealed the prevalent human exposure to Pb and increased risk of Pb toxicity. Once ingested by human, 95% of absorbed Pb is accumulated into erythrocytes and erythrocytes are known to be a prime target for Pb toxicity. Most of the studies were however, focused on Pb^2+^ whereas the effects of Pb^4+^, another major form of Pb on erythrocytes are poorly understood yet. In this study, we investigated and compared the effects of Pb^4+^, Pb^2+^ and other heavy metals on procoagulant activation of erythrocytes, an important factor for the participation of erythrocytes in thrombotic events in an effort to address the cardiovascular toxicity of Pb^4+^. Freshly isolated erythrocytes from human were incubated with Pb^4+^, Pb^2+^, Cd^2+^ and Ag^+^ and the exposure of phosphatidylserine (PS), key marker for procoagulant activation was measured using flow cytometry. As a result, while Cd^2+^ and Ag^+^ did not affect PS exposure, Pb^4+^ and Pb^2+^ induced significantly PS exposure in a dose-dependent manner. Of a particular note, Pb^4+^ induced PS exposure with a similar potency with Pb^2+^. PS bearing microvesicle (MV), another important contributor to procoagulant activation was also generated by Pb^4+^. These PS exposure and MV generation by Pb^4+^ were well in line with the shape change of erythrocyte from normal discocytes to MV shedding echinocytes following Pb^4+^ treatment. Meanwhile, nonspecific hemolysis was not observed suggesting the specificity of Pb^4+^-induced PS exposure and MV generation. These results indicated that Pb^4+^ could induce procoagulant activation of erythrocytes through PS exposure and MV generation, suggesting that Pb^4+^ exposure might ultimately lead to increased thrombotic events.

## INTRODUCTION

Lead (Pb) is a widely distributing environmental element and extensively used in human life for a long time. Pb exists in 4 oxidized states from +1 to +4 and of these, +2 and +4 constitutes a major portion of naturally occurring Pb. Human is exposed to Pb through industrial activities as well as Pb contaminated food, water and Pb applied consumer products. Normal blood lead level (BLL) is below 5 μg/dl and Pb poisoning is defined when BLL is more than 10 μg/dl, substantially getting stricter from the guideline (> 60 μg/dl) of 1970s (CDCP, 1997). This trend is from the recent discoveries of profound and severe toxicity of Pb to human, especially to the younger children.

Once absorbed into systemic blood flow, more than 95% of Pb is associated with erythrocytes mainly in cellular membrane or hemoglobin (Goyer *et al.*, 2001). Therefore, erythrocytes are considered as a prime target for Pb toxicity (Battistini *et al.*, 1971). Pb denatures cellular protein and lipid components of erythrocytes (Fukumoto *et al.*, 1983) and impairs the synthesis of hemoglobin (Monteiro *et al.*, 1989; Waldron, 1966). In addition, a variety of toxic responses can be induced in erythrocytes by Pb such as lipid peroxidation and oxidative stress (Gurer *et al.*, 1998; Quinlan *et al.*, 1988), inhibition of superoxide dismutase activities and depletion of glutathione level (Ito *et al.*, 1985; Sugawara *et al.*, 1991). Pb exposure shortens the life span of erythrocytes and resultantly, anemia can be induced (Iavicoli *et al.*, 2003; Waldron, 1966). It also brings about erythrocyte shape changes from discocytic normocytes to abnormal echinocytes (Kempe *et al.*, 2005).

Erythrocytes constitute a major cell population in blood, working essentially as the oxygen carriers. Recently, it has been revealed that through the interaction with vascular endothelial cells, platelets and leukocytes, erythrocytes can actively participate in thrombosis and haemostasis (Marcus, 1990). Erythrocytes can express phosphatidylserine (PS) on the cellular membrane through the disruption of lipid asymmetry. PS exposure can be a phagocytic signal to the macrophages (Fadok *et al.*, 1993) and more importantly, can work as a facilitator of procoagulation pathways by providing a site for the assembly of the prothrombinase and tenase complex, leading to thrombin generation and clotting (Zwaal, 1978; Zwaal *et al.*, 1977).

Previously, we have shown that $Pb^{2+}$ induces procoagulant activation of erythrocytes through PS exposure (Shin *et al.*, 2007). However, there was no information available about the effects of $Pb^{4+}$ on erythrocytes. In this study, we investigated and compared the effects of $Pb^{4+}$ and $Pb^{2+}$ on erythrocytes in terms of shape changes and procoagulation activation in an effort to enlighten the potential cardiovascular toxicity of $Pb^{4+}$.

## MATERIALS AND METHODS

***Materials.*** Lead(IV) acetate, lead(II) acetate, $CdCl_2$, $AgNO_3$, dimethyl sulfoxide (DMSO), $CaCl_2$, ethylenediaminetetraacetic acid (EDTA), $KH_2PO_4$, NaCl, $Na_2HPO_4$, KCl, Tris/HCl, $MgCl_2$, $NaH_2PO_4$, glutaraldehyde, ethanol, osmium tetroxide were obtained from Sigma chemical Co. (St. Louis, MO). Fluorescein-isothiocyanate (FITC)-labeled annexin V (annexin V-FITC) was from Pharmingen (San Diego, CA) and phycoerythrin-labeled monoclonal antibody against human glycophorin A (anti-glycophorin A-RPE) was purchased from Dako Cytomation (Glostrub, Denmark).

***Preparation of erythrocytes.*** With an approval from the Ethics Committee of Health Service Center at Seoul National University, human blood was obtained from healthy male donors (18~25 years old) using a vacutainer with acid citrate dextrose (ACD) and a 21-gauge needle (Becton Dickinson, Franklin Lakes, NJ) on the day of each experiment. Platelet rich plasma and buffy coat were removed by aspiration after centrifugation at 200 g for 15 min. Packed erythrocytes were washed 3 times with phosphate buffered saline (PBS: 1 mM $KH_2PO_4$, 154 mM NaCl, 3 mM $Na_2HPO_4$, pH 7.4) and once with Tris buffer (TBS: 15 mM Tris-HCl, 150 mM NaCl, 5 mM KCl, 2 mM $MgCl_2$, pH 7.4). Washed erythrocytes were resuspended in TBS to a cell concentration of $5 \times 10^7$ cells/ml and final $CaCl_2$ concentration was adjusted to 1 mM prior to use.

***Detection of hemolysis.*** For detecting hemolysis, after erythrocytes were treated with vehicle (DMSO) or lead(IV) acetate for 4 hr at 37°C, the incubation was stopped by centrifugation (1 min at 12,000 g) and supernatants were harvested. Hemolysis of control erythrocytes in distilled water was defined as complete hemolysis and the extent of hemolysis was determined spectrophotometrically at 540 nm. The blank value was determined with vehicle (DMSO), respectively. Sample values were expressed as the percentage of complete hemolysis.

***Flow cytometric analysis of phosphatidylserine exposure and microvesicle generation.*** Annexin V-FITC was used as a marker for phosphatidylserine (PS) detection, while anti-glycophorin A-RPE was used as an identifier of erythrocytes. Negative controls for annexin V binding were stained with annexin V-FITC in the presence of EDTA 2.5 mM instead of $CaCl_2$ 2.5 mM. Samples were analyzed on the flow cytometer FACScalibur™ (Becton Dickinson, San Jose, CA) equipped with argon-ion laser emitting at 488 nm. The light scatter and fluorescence channels were set on log scale. Data from 10,000 events were collected and analyzed using CellQuest™ Pro software.

***Microscopic observation using scanning electron microscopy.*** After fixation with 2% glutaraldehyde solution for 1 hr at 4°C, the erythrocytes were centrifuged and washed 3 times with PBS, and followed by post-fixation with 1% osmium tetroxide for 30 min at room temperature. After washing with PBS several times, the samples were dehydrated serially with 50, 75, 90 and 100% ethanol. After drying and coating with gold, the images were observed on scanning electron microscope (JEOL, Japan).

***Statistical analysis.*** The means and standard errors of means were calculated for all treatment groups. The data were subjected to one-way analysis of variance (ANOVA) followed by Duncan's multiple range test or student t-test to determine which means were significantly different from the control. In all cases, a $p$ value of $< 0.05$ was used to determine significant differences.

## RESULTS

***Effects of $Pb^{4+}$ and other metal ions on phosphatidylserine exposure.*** Phosphatidylserine (PS) expo-

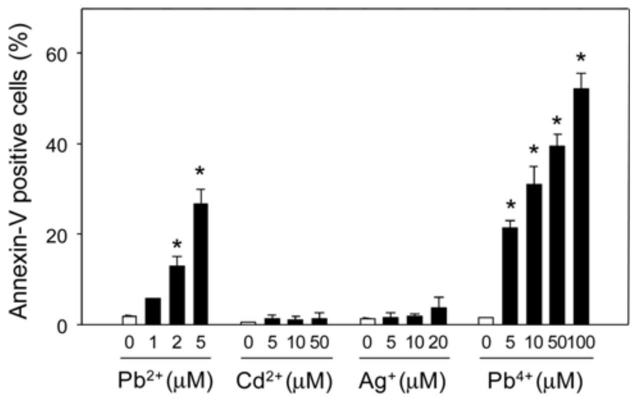

**Fig. 1.** Effects of heavy metals on PS exposure in human red blood cells. After RBCs were treated with DW (vehicle) or lead(II) acetate for 4 hrs; TBS (vehicle) or $CdCl_2$ for 1 hr; DMSO (vehicle) or $AgNO_3$ for 1 hr; DMSO (vehicle) or lead(IV) acetate for 4 hr at 37°C, the extent of PS exposure was measured by flow cytometry. Values are mean ± SEM of three to four independent experiments. * represents significant difference from control ($p < 0.05$).

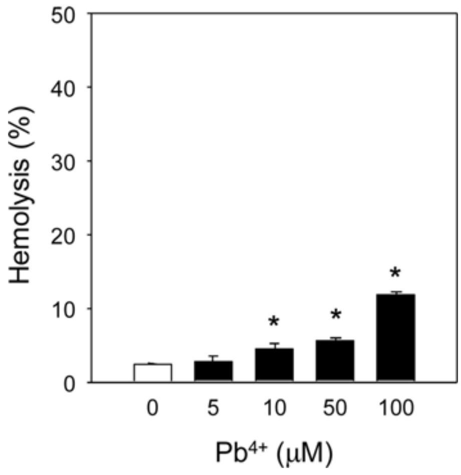

**Fig. 3.** Effects of $Pb^{4+}$ on hemolysis in human red blood cells. After RBCs were treated with DMSO (vehicle) or lead(IV) acetate for 4 hr at 37°C, the extent of hemolysis was measured. Values are mean ± SEM of three to four independent experiments. * represents significant difference from control ($p < 0.05$).

sure on outer cellular membrane is an important marker for the procoagulant activation of erythrocytes. PS exposure was measured using annexin V-FITC by flow cytometry analysis after erythrocytes were incubated with $Pb^{4+}$, $Pb^{2+}$, $Cd^{2+}$ and $Ag^{+}$ or vehicle at 37°C. As a result, PS exposure was significantly increased by $Pb^{2+}$ and $Pb^{4+}$ while no effects could be found with $Cd^{2+}$ or $Ag^{+}$ (Fig. 1). Conspicuously, a similar potency for PS exposure could be observed with $Pb^{4+}$ and $Pb^{2+}$ at 5 μM, indicating that $Pb^{4+}$ and $Pb^{2+}$ might show similar vascular toxic effect. PS exposure was induced in a concentration and time dependent manner by $Pb^{4+}$ (Fig. 2), suggesting that higher and stronger procoagulation activation might be caused by chronic exposure to $Pb^{4+}$.

***Effects of $Pb^{4+}$ on hemolysis and microvesicle generation.*** To investigate whether nonspecific hemolysis might be involved in $Pb^{4+}$-induced PS exposure, extent of hemolysis was measured following the incuba-

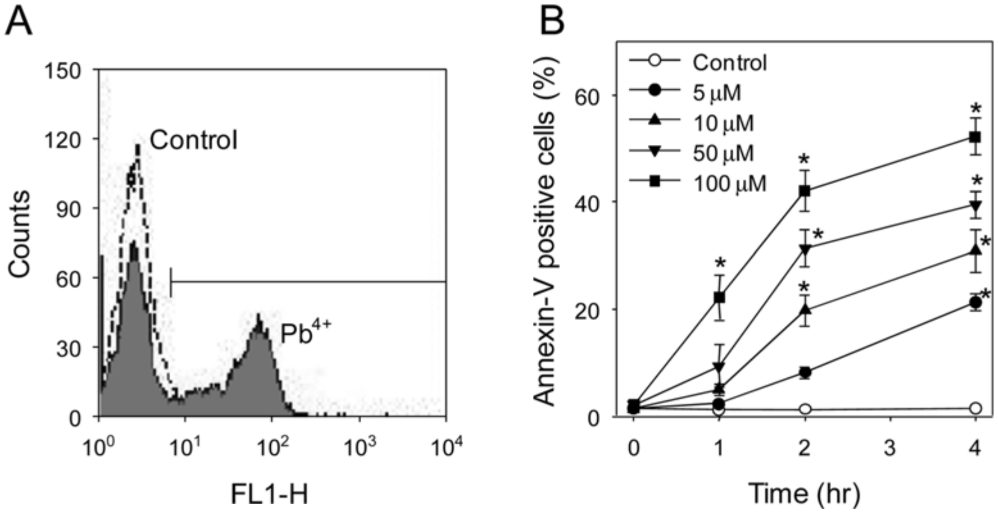

**Fig. 2.** Effects of $Pb^{4+}$ on phosphatidylserine exposure in human red blood cells. After RBCs were treated with DMSO (vehicle) or lead(IV) acetate for 4 hr at 37°C, the extent of PS exposure was measured. (A) the representative fluorescence histogram, (B) time and concentration dependent increase of PS exposure is shown. Values are mean ± SEM of three to four independent experiments. * represents significant difference from control ($p < 0.05$).

tion with $Pb^{4+}$. As a result, in contrast to the potent PS exposure, $Pb^{4+}$ only induced a minimal extent of hemolysis less than 10% up to 100 μM, indicating that contribution from nonspecific hemotoxicity was negligible (Fig. 3).

In addition to PS exposure on erythrocyte mem-

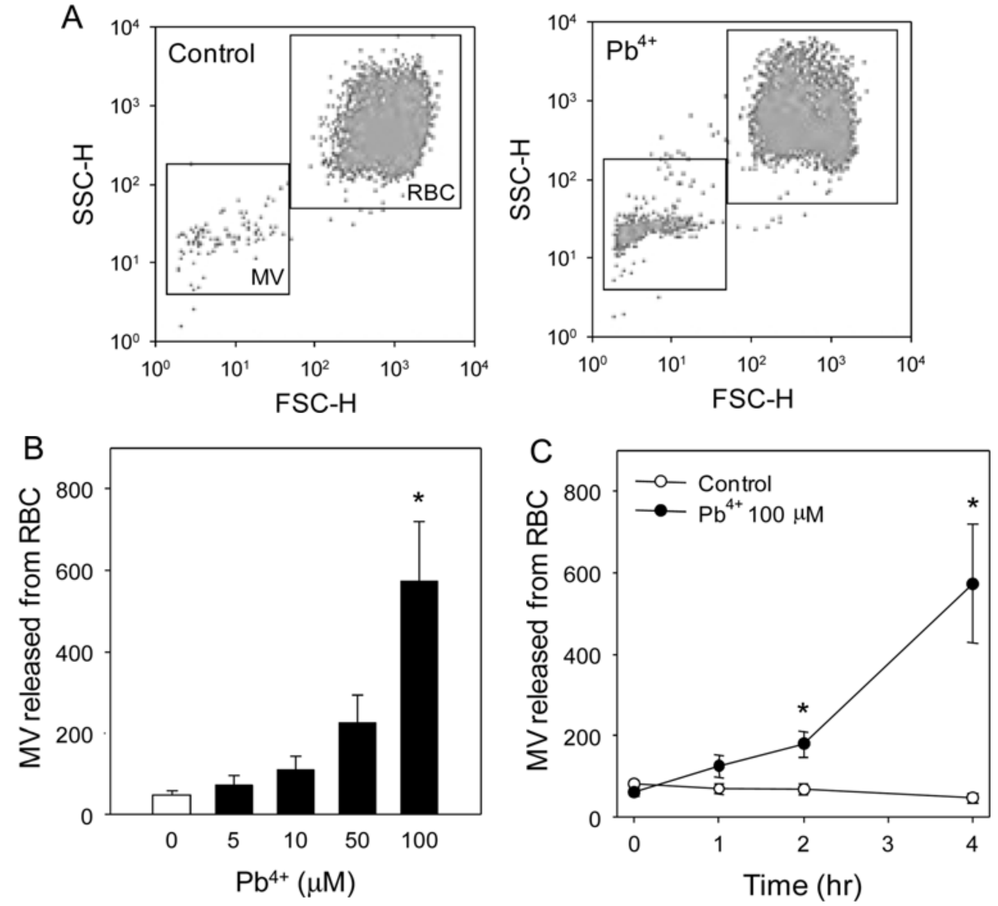

**Fig. 4.** Effects of $Pb^{4+}$ on microvesicle generation in human red blood cells. After RBCs were treated with DMSO (vehicle) or lead(IV) acetate for 4 hr at 37°C, the extent of MV generation was measured. (A) the representative dot plot, (B) concentration and (C) time dependent increase of MV generation is shown. Values are mean ± SEM of three to four independent experiments. * represents significant difference from control ($p < 0.05$).

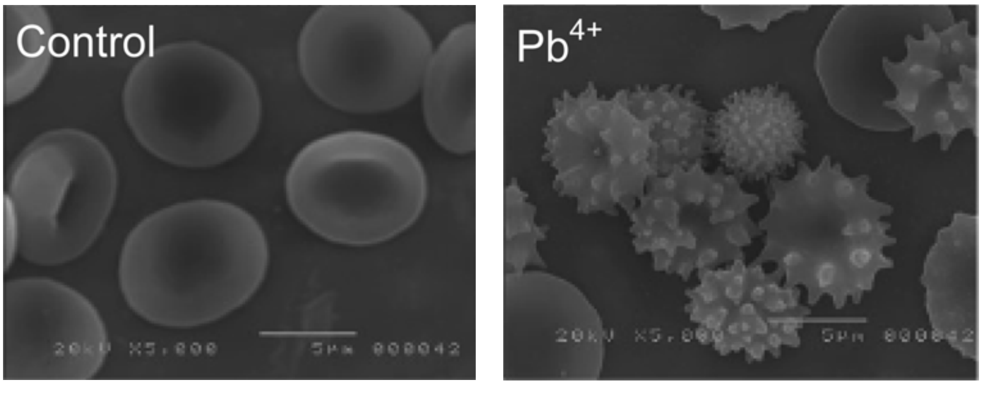

**Fig. 5.** Effects of $Pb^{4+}$ on shape changes in human red blood cells. After RBCs were treated with DMSO (vehicle) or 100 μM lead(IV) acetate for 4 hours at 37°C. The cells were fixed and the morphological changes were examined under scanning electron microscope. Representative microscopic photograph was shown here (original magnification: × 3,500).

brane, PS bearing microvesicles (MV) shed from erythrocytes could contribute to the enhancement of procoagulant activity. To investigate whether $Pb^{4+}$ could induce MV generation in erythrocytes, MV was identified with annexin V-FITC and anti-glycophorin A-RPE through forward scatter in flow cytometry. Although with a less potency than PS exposure, $Pb^{4+}$ increased significantly the generation of MV from erythrocytes significantly in a concentration and time-dependent manner (Fig. 4).

***Effects of $Pb^{4+}$ on morphological change in RBCs.*** PS exposure and MV generation often accompany shape changes in erythrocytes. Especially, MV generation is closely related with abnormal shapes of erythrocytes like echinocytes, spherocytes or stomatocytes. To examine the changes in the shape of erythrocytes by $Pb^{4+}$, erythrocytes were observed with scanning electron microscope following the exposure to $Pb^{4+}$. As shown in Fig. 5, $Pb^{4+}$ treatment induced abnormal shape changes in erythrocytes from normal discocytic shapes to echinocytes, further confirming the $Pb^{4+}$ induced MV generation and PS exposure.

## DISCUSSION

In the current study, we demonstrated that $Pb^{4+}$ could induce phosphatidylserine (PS) exposure and microvesicle (MV) generation in erythrocytes. $Pb^{4+}$-induced PS exposure and MV generation were increased in a concentration and time-dependent manner and confirmed to be not related to nonspecific hemolysis. Along with PS exposure and MV generation, $Pb^{4+}$ induced abnormal shape changes from normal discocytic shapes into echinocytic shapes. These results suggest the potential roles of procoagulant activation of erythrocytes in $Pb^{4+}$-associated cardiovascular events.

As shown in Fig. 2 and 4, $Pb^{4+}$ induced PS exposure and MV generation potently. PS exposed on erythrocyte membrane provides a site for the assembly of tenase and prothrombinase during blood coagulation, facilitating the conversion of factor X and prothrombin to active factor Xa and thrombin (Kalafatis *et al.*, 1994; Mann *et al.*, 1990). The activated factor Xa and thrombin lead to the acceleration of blood coagulation, that is, procoagulant activation. Erythrocytes from many pathological states such as sickle cell anemia and thalassemia express increased procoagulant activity (Helley *et al.*, 1996; Martin *et al.*, 1995). In addition, endogenous thrombogenic substances like arachidonic acid, lysophosphatidic acid and thromboxane are known to be able to induce procoagulant activation of erythrocytes (Chung *et al.*, 2007; Valles *et al.*, 2002). MV generated from erythrocyte membrane could harbor PS on its outer membrane and also has a procoagulant activity (Franck *et al.*, 1985), indicating that PS exposure and MV generation might ultimately lead to increased risk of cardiovascular diseases (Zwaal, 1978; Zwaal *et al.*, 1977).

In this study, the procoagulant activation of erythrocytes could be induced by $Pb^{4+}$ at relatively low concentrations (5 μM) which was similar with the active concentrations of $Pb^{2+}$ (2~5 μM) (Fig. 1). Considering that blood lead level for lead poisoning is 10 μg/dl (0.5 μM), the active concentrations of $Pb^{4+}$ are well below clinically relevant exposure levels of lead.

Previously, it was reported that Pb can induce abnormal shape changes in erythrocytes (Baranowska-Bosiacka *et al.*, 2003), however, there has been no information on MV generation by Pb (Shin *et al.*, 2007). We also could not find MV generation with $Pb^{2+}$. In the current study, we could demonstrate that $Pb^{4+}$ could induce MV generation as well as abnormal shape changes. These results further support that $Pb^{4+}$ could be a more potent procoagulant activator of erythrocytes indicating that $Pb^{4+}$ might also be important for lead-associated cardiovascular diseases.

Previously, we have shown that PS exposure by $Pb^{2+}$ was mediated by intracellular calcium increase and ATP depletion (Shin *et al.*, 2007). ATP depletion and calcium increase affect aminophospholipid translocases which maintain membrane lipid asymmetry. More specifically, ATP depletion and calcium increase impair the activity of flippase which recover exposed PS into inner membrane while scramblase, an enzyme scrambling lipid asymmetry is activated by calcium increase. Although further confirmatory studies are necessary, it is expected that PS exposure by $Pb^{4+}$ would also undergo ATP depletion and calcium increase. However, involvement of another novel pathway might not be excluded since $Pb^{4+}$ could induce MV generation of which aspect could not be observed with $Pb^{2+}$.

In conclusion, this study demonstrated that micromolar concentrations of $Pb^{4+}$ could induce procoagulant activation of erythrocytes through PS exposure and PS bearing MV generations. These $Pb^{4+}$-induced procoagulant activation of erythrocytes might lead to increased blood coagulation and thrombosis, ultimately. Along with the prevalent and global lead contamination problem, this study suggests that more efforts should be devoted into the research on $Pb^{4+}$-induced cardiovascular toxicity.

## ACKNOWLEDGMENTS

This work was supported by National Research Foundation of Korea Grant funded by the Korean Government (KRF-2006-E00162).
